# Coenzyme Q_10_ Reduces Ethanol-Induced Apoptosis in Corneal Fibroblasts

**DOI:** 10.1371/journal.pone.0019111

**Published:** 2011-04-27

**Authors:** Chun-Chen Chen, Shiow-Wen Liou, Chi-Chih Chen, Wen-Chung Chen, Fung-Rong Hu, I-Jong Wang, Shing-Jong Lin

**Affiliations:** 1 Department of Ophthalmology, Taipei City Hospital Renai Branch, Taipei, Taiwan; 2 Institute of Clinical Medicine, National Yang-Ming University, Taipei, Taiwan; 3 Cardiovascular Research Center, National Yang-Ming University, Taipei, Taiwan; 4 Division of Cardiology, Department of Internal Medicine, Taipei Veterans General Hospital, Taipei, Taiwan; 5 Department of Medical Research and Education, Taipei Veterans General Hospital, Taipei, Taiwan; 6 Department of Ophthalmology, National Taiwan University Hospital, Taipei, Taiwan; 7 Taipei Medical University, Taipei, Taiwan; University of South Florida College of Medicine, United States of America

## Abstract

Dilute ethanol (EtOH) is a widely used agent to remove the corneal epithelium during the modern refractive surgery. The application of EtOH may cause the underlying corneal fibroblasts to undergo apoptosis. This study was designed to investigate the protective effect and potential mechanism of the respiratory chain coenzyme Q_10_ (CoQ_10_), an electron transporter of the mitochondrial respiratory chain and a ubiquitous free radical scavenger, against EtOH-induced apoptosis of corneal fibroblasts. Corneal fibroblasts were pretreated with CoQ_10_ (10 µM) for 2 h, followed by exposure to different concentrations of EtOH (0.4, 2, 4, and 20%) for 20 s. After indicated incubation period (2–12 h), MTT assay was used to examine cell viability. Treated cells were further assessed by flow cytometry to identify apoptosis. Reactive oxygen species (ROS) and the change in mitochondrial membrane potential were assessed using dichlorodihydrofluorescein diacetate/2′,7′-dichlorofluorescein (DCFH-DA/DCF) assays and flow-cytometric analysis of JC-1 staining, respectively. The activity and expression of caspases 2, 3, 8, and 9 were evaluated with a colorimetric assay and western blot analysis. We found that EtOH treatment significantly decreased the viability of corneal fibroblasts characterized by a higher percentage of apoptotic cells. CoQ_10_ could antagonize the apoptosis inducing effect of EtOH. The inhibition of cell apoptosis by CoQ_10_ was significant at 8 and 12 h after EtOH exposure. In EtOH-exposed corneal fibroblasts, CoQ_10_ pretreatment significantly reduced mitochondrial depolarization and ROS production at 30, 60, 90, and 120 min and inhibited the activation and expression of caspases 2 and 3 at 2 h after EtOH exposure. In summary, pretreatment with CoQ_10_ can inhibit mitochondrial depolarization, caspase activation, and cell apoptosis. These findings support the proposition that CoQ_10_ plays an antiapoptotic role in corneal fibroblasts after ethanol exposure.

## Introduction

The excimer laser is an effective tool, not only for correcting myopic refractive error, but also for treating various types of anterior corneal pathology [1]. The success of excimer laser refractive surgery, including photorefractive surgery (PRK) and laser subepithelial keratomileusis (LASEK), depends on thorough preoperative assessment and attention to intraoperative details. However, myopic regression and prolonged visual rehabilitation are two common consequences of these surface ablations [2]. Although individual differences often result in variable corneal wound healing after refractive surgery, the methods of corneal epithelial removal used in these refractive procedures can also affect the occurrence of these undesirable outcomes [3]. The application of diluted ethanol (EtOH) is one of the most widely used methods to remove the epithelium during the procedures of surface ablation [2], [4]. Ethanol delamination of the corneal epithelium consistently results in very smooth cleavage at the level of the hemidesmosomal attachments, including the superficial lamina lucida, and is used to create a smooth stromal surface for further surface ablation [5].

The corneal stromal cell apoptosis has been well-characterized as an early initiating event of the corneal healing response during refractive surgery [6], [7]. It triggers subsequent cellular processes that include bone marrow-derived cell infiltration, proliferation of residual corneal stromal cells, and in some circumstances, generation of myofibroblasts [6], [8]. Ethanol may induce apoptosis in a variety of tissues, including corneal epithelial cells [9], [10], corneal keratocytes [11], Liver [12], and brain [13]. Adverse effects of alcohol on corneal fibroblasts have been reported, including apoptotic changes in the anterior stromal keratocytes, especially around the epithelial flap margin, and these can result in cell loss after treatment [7], [14], [15]. In the cornea, the apoptosis of corneal fibroblasts induced by ethanol plays an important role in the chemokinetics of corneal wound healing [16], myofibroblast transformation [17], and corneal neovascularization [18]. Modulation of cell apoptosis in the corneal stroma is thought to be critical for ideal refractive surgery, with early stromal cell apoptosis regarded as a promising target for controlling later events in the wound healing cascade [8]. To date, pharmacological efforts to control early corneal stromal cell apoptosis have not been successful, but research is ongoing to identify agents that can regulate this phenomenon.

Recent studies have demonstrated that free radical formation increases after excimer laser surgery [19], [20], [21]. The exposure of cells to ethanol can alter the cytosolic and mitochondrial redox states and disrupt the functions of various metabolic pathways, which also increase free radical formation [21]. Antioxidants and free radical scavengers have been reported to protect the cornea from the harmful effects of free radicals [22], [23]. The protective effects of ascorbic acid and vitamin E after PRK have been demonstrated in previous studies [23], [24]. Ubiquinone Q_10_ (coenzyme Q_10_, CoQ_10_) is a well-known electron transporter in complexes I (NADH–ubiquinone oxidoreductase), II (succinate–ubiquinone oxidoreductase), and III (ubiquinone–cytochrome *c* oxidoreductase) of the mitochondrial respiratory chain [25], [26]. CoQ_10_ is also a ubiquitous free radical scavenger that inhibits cellular apoptosis [25], [27], [28], [29], [30]. Brancato *et al* have demonstrated that CoQ_10_ reduces the number of apoptotic keratocytes produced in response to excimer laser irradiation to a much greater extent than do other free radical scavengers, such as ascorbic acid and vitamin E [31]. CoQ_10_ also plays a role in the control of mitochondrial transition pore opening, which is involved in apoptosis [32].

In this study, we hypothesized that CoQ_10_ pretreatment increases cell viability and reduces cell apoptosis in corneas exposed to ethanol during refractive surgery. Therefore, we evaluated the effects of CoQ_10_ pretreatment on ethanol-exposed corneal fibroblasts *in vitro*.

## Results

### Effect of coenzyme Q_10_ on ethanol-treated corneal fibroblast viability

**Figure 1:**
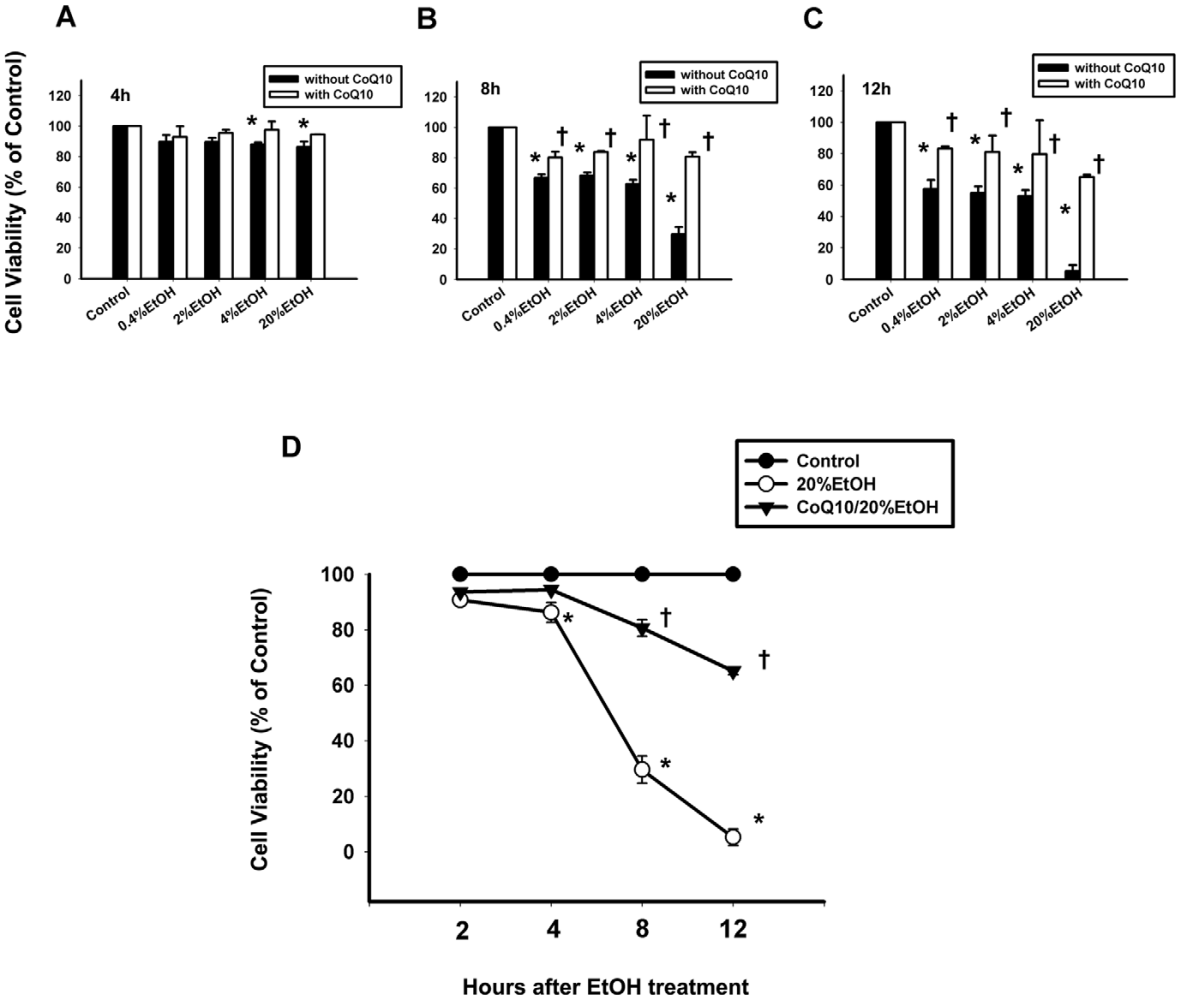
The influence of CoQ_10_ on EtOH induced cell death in corneal fibroblasts. The cells were pretreated with 10 µM CoQ_10_ for 2 h. Thereafter, EtOH (A–D: 0.4%, 2%, 4%, 20%, E: 20%) at indicated concentrations were added. After incubation period (2, 4, 8, or 12 h), the cell viability was determined by MTT assay. Data represent the results of three independent experiments (means ± SD; *P<0.05 compared with the control group; †*P*<0.05 compared with the EtOH group).

To assess the changes in cell viability after EtOH exposure, we evaluated its dose- and time-dependent effects on cultured corneal fibroblasts with or without CoQ_10_ pretreatment. At lower concentrations of EtOH, i.e., <20%, EtOH exposure alone decreased cell viability at indicated concentrations (Table 1). Cell viability was significantly reduced at 4 h in the 4% EtOH group, at 8, 12 h in 0.4%, 2%, and 4% EtOH groups. The pretreatment with CoQ_10_ significantly increased cell viability at 8, 12 h in 0.4%, 2%, and 4% CoQ_10_/EtOH groups (Table 1 and Figure 1). Whereas, at the higher concentration of EtOH, i.e., 20%, the percentages of cell viability measured at 2, 4, 8 and 12 h after treatment with 20% EtOH for 20 s were as follows: 2 h, 90.71±0.12%; 4 h, 86.22±3.56%; 8 h, 29.58±4.78%; 12 h, 5.23±3% (one-way ANOVA, *P*<0.05; Figure 1D). The viability of the EtOH-exposed (20%, 20 s) cells was significantly reduced compared with that of the control group at all time points (2 h, *P* = 0.02; 4 h, *P* = 0.03; 8 h, *P*<0.0001; 12 h, *P*<0.0001). The percentage of cell viability was increased in cells pretreated with CoQ_10_ after EtOH exposure (2 h, 93.6±0.57%; 4 h, 94.43±0.12%; 8 h, 80.58±2.98%; 12 h, 65.11±1.39%). The protective effect of CoQ_10_ was significant at 8 and 12 h after EtOH treatment (2 h, *P* = 0.1; 4 h, *P* = 0.1; 8 h, *P* = 0.01; 12 h, *P* = 0.002).

**Table 1:** Effect of CoQ_10_ on cell viability measured by the MTT assay in corneal fibroblasts.

Group	Incubation interval (h)	Cell viability a (% of control)	P value vs control	P value vs EtOH group
Control	4	100		
0.4% EtOH	4	89.65±4.45%	0.08	
CoQ_10_/0.4%EtOH	4	92.85±7%		0.6
2% EtOH	4	89.60±2.69%	0.06	
CoQ_10_/2%EtOH	4	95.40±2.12%		0.09
4% EtOH	4	87.95±1.34% b	0.01	
CoQ_10_/4%EtOH	4	97.50±5.51%		0.05
Control	8	100		
0.4% EtOH	8	66.68±2.31% b	0.002	
CoQ_10_/0.4%EtOH	8	80.12±3.81% c		0.01
2% EtOH	8	68.31±1.96% b	0.001	
CoQ_10_/2%EtOH	8	83.64±0.81% c		0.009
4% EtOH	8	62.62±2.84% b	0.003	
CoQ_10_/4%EtOH	8	91.71±15.97% c		0.02
Control	12	100		
0.4% EtOH	12	57.47±5.76% b	0.009	
CoQ_10_/0.4%EtOH	12	83.25±1.4% c		0.025
2% EtOH	12	54.98±4.08% b	0.004	
CoQ_10_/2%EtOH	12	81.03±10.5% c		0.032
4% EtOH	12	52.91±3.85% b	0.003	
CoQ_10_/4%EtOH	12	79.61±21.65% c		0.04

a Cells were pretreated with 10 µM of CoQ_10_ before different concentrations of EtOH (0.4%, 2%, 4%) exposure for 20 s and then cell viability was measured by MTT assay after 4, 8, and 12 h.

b P<0.05, compared with control group.

c P<0.05, compared with EtOH group.

### Effects of coenzyme Q_10_ on the apoptosis of ethanol-treated corneal fibroblasts

**Figure 2:**
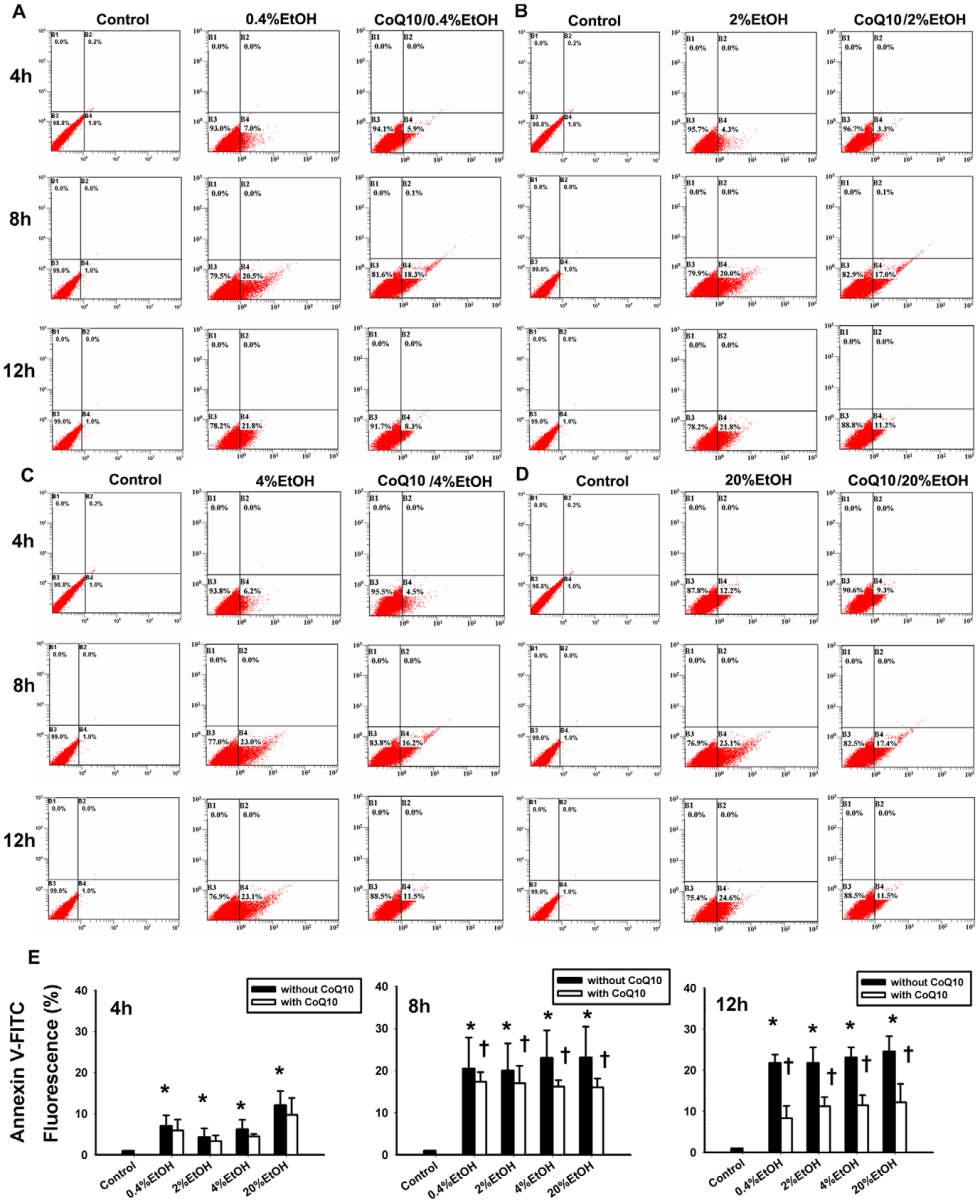
Pretreatment of corneal fibroblasts for 2 h with 10 µM CoQ_10_ inhibited the cell apoptosis induced by EtOH exposure. (A–D) Cells were pretreated with or without CoQ_10_, followed by different EtOH treatment (0.4%, 2%, 4%, and 20%) for 20 s. The apoptotic cells were stained with annexin V-PI for flow-cytometric analysis at 4, 8, and 12 h. (E) The bar diagram shows the comparison of the relative fluorescence of annexin V–FITC fluorescence intensity at different times. Data represent the results of three independent experiments (means ± SD; *P<0.05 compared with the control group; †*P*<0.05 compared with the EtOH group).

To determine the effects of CoQ_10_ on programmed cellular death after EtOH exposure, we analyzed cell apoptosis with annexin V-FITC and PI double staining methods after incubation for 4, 8, or 12 h (Figure 2). EtOH exposure induced remarkable number of apoptotic cells upon 4, 8, and 12 h of incubation period. Treatment of EtOH at the concentrations of 0.4–4% showed a significant increase in apoptotic cells over the untreated cells after 4, 8, and 12 h. The pretreatment with CoQ_10_ significantly decreased the percentage of apoptotic cells induced by EtOH at 8 and 12 h time points (Table 2 and Figure 2A–2C). As shown in Figure 2D–2E, the percentages of annexin V–FITC-positive cells after EtOH exposure (20%, 20 s) were as follows: 4 h, 15.47±3.4%; 8 h, 21.03±9.0%; 12 h, 29.8±2.2% (one-way ANOVA, *P*<0.05). Cell apoptosis was significantly increased after EtOH exposure compared with that in the control group at all time points (4 h, *P* = 0.01; 8 h, *P* = 0.01; 12 h, *P* = 0.02). The percentages of EtOH-induced apoptosis were also attenuated by pretreatment with CoQ_10_ (4 h, 12.87±4.1%; 8 h, 14.63±2.11%; 12 h, 17.62±6.48%). CoQ_10_ pretreatment significantly reduced cell apoptosis at 8 and 12 h after EtOH exposure (4 h, *P* = 0.1; 8 h, *P* = 0.01; 12 h, *P*<0.0001).

**Table 2:** Effect of CoQ_10_ on cell apoptosis induced by EtOH in corneal fibroblasts.

Group	Incubation period (h)	Apoptotic rate a (%)	P value vs control	P value vs EtOH group
Control	4	1		
0.4% EtOH	4	7.0±2.6 b	0.02	
CoQ_10_/0.4%EtOH	4	5.9±2.7		0.06
2% EtOH	4	4.3±2.1 b	0.03	
CoQ_10_/2%EtOH	4	3.3±1.4		0.09
4% EtOH	4	6.2±2.3 b	0.02	
CoQ_10_/4%EtOH	4	4.5±0.6		0.07
Control	8	1		
0.4% EtOH	8	20.5±7.34 b	0.002	
CoQ_10_/0.4%EtOH	8	17.3±2.31 c		0.008
2% EtOH	8	20.0±6.43 b	0.006	
CoQ_10_/2%EtOH	8	17.0±4.12 c		0.005
4% EtOH	8	23.0±6.50 b	0.001	
CoQ_10_/4%EtOH	8	16.2±1.50 c		0.003
Control	12	1		
0.4% EtOH	12	21.8±2.0 b	0.005	
CoQ_10_/0.4%EtOH	12	8.3±3.0 c		0.002
2% EtOH	12	21.8±3.76 b	0.005	
CoQ_10_/2%EtOH	12	11.2±2.25 c		0.004
4% EtOH	12	23.1±2.45 b	0.003	
CoQ_10_/4%EtOH	12	11.5±2.43 c		0.001

a Cells were pretreated with 10 µM of CoQ_10_ before different concentrations of EtOH (0.4%, 2%, 4%) exposure for 20 s and then cell apoptosis was measured by flow cytometry after 4, 8, and 12 h.

b P<0.05, compared with control group.

c P<0.05, compared with EtOH group.

### Effects of coenzyme Q_10_ on the ROS induced by ethanol treatment

**Figure 3:**
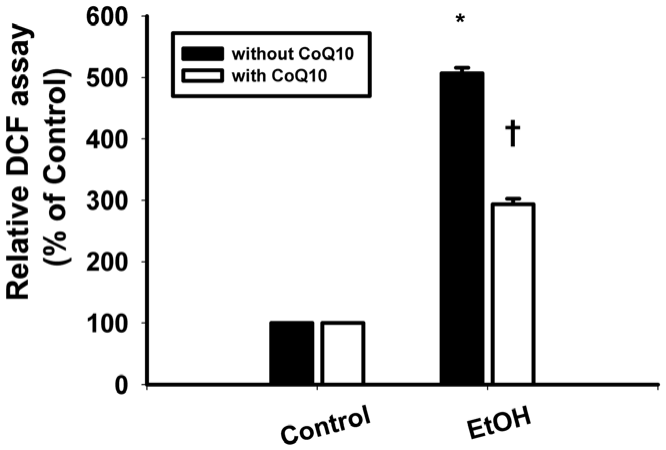
Pretreatment of corneal fibroblasts for 2 h with 10 µM CoQ_10_ reduced the elevated reactive oxygen species (ROS) levels induced by EtOH exposure. ROS levels in cells pretreated with or without CoQ_10_, followed by EtOH treatment (20%, 20 s), were measured by analyzing the DCF intensity after 30 min. Data represent the results of three independent experiments performed in triplicate (means ± SD; *P<0.05 compared with the control group; †*P*<0.05 compared with the EtOH group).

The results of the DCF assay indicated that EtOH exposure (20%, 20 s) caused an increase in the intracellular levels of ROS. The relative DCF fluorescence intensities measured after EtOH exposure at 30, 60, 90, and 120 min were as follows: 30 min, 506.5±9.19%; 60 min, 305±7.07%; 90 min, 142.5±10.6%; 120 min, 126.0±8.49%. EtOH exposure caused an increase of at least fivefold in intracellular ROS levels at min. CoQ_10_ pretreatment reduced the ROS levels in EtOH-exposed cells at all time points (30 min, 293.5±9.19%; 60 min, 198±2.83%; 90 min, 115±7.07%; 120 min, 107±9.09%; Figure 3). The intracellular ROS levels were significantly lower in cells pretreated with CoQ_10_ at 30, 60, 90, and 120 min after exposure to EtOH (30 min, *P*<0.0001; 60 min, *P* = 0.01; 90 min, *P* = 0.02; 120 min, *P* = 0.02).

### Effects of coenzyme Q_10_ on ethanol-treated cellular mitochondrial membrane potential

**Figure 4:**
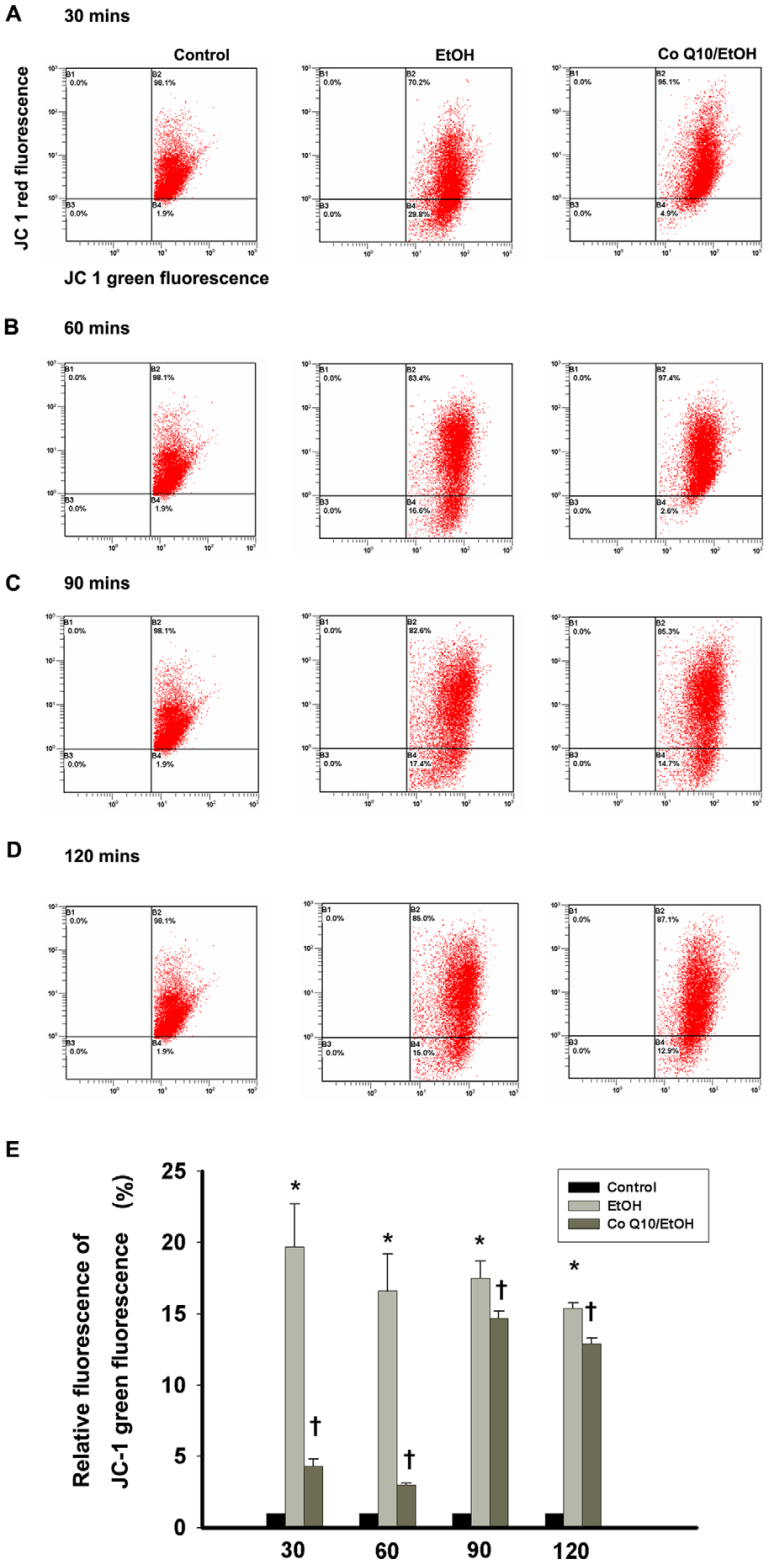
Pretreatment of corneal fibroblasts for 2 h with 10 µM CoQ_10_ reduced the change in mitochondrial membrane potential induced by EtOH exposure. The mitochondrial membrane potential transition (MPT) was determined in cells pretreated with or without CoQ_10_, followed by EtOH treatment (20%, 20 s), after intervals of (A) 30, (B) 60, (C) 90, and (D) 120 min. Loss of mitochondrial membrane potential was demonstrated by the change in JC-1-derived fluorescence from red (high potential as JC-1 aggregates) to green (low potential as JC-1 monomer). (E) The bar diagram shows the relative JC-1 green fluorescence after normalization to control at all intervals. Data represent the results of three independent experiments performed in triplicate (means ± SD; *P<0.05 compared with the control group; †*P*<0.05 compared with the EtOH group).

The changes in the mitochondrial membrane potential of EtOH-exposed (20%, 20 s) cells were determined by JC-1 staining. The extranuclear mitochondria in the cells of the control group primarily emitted red fluorescence, whereas the mitochondria undergoing mitochondrial membrane potential transition (MPT), which were located diffusely throughout the whole cells, were detected as green particles. Flow-cytometric analysis of MPT demonstrated that the relative green signals (Figure 4) of the EtOH-exposed cells at 30, 60, 90, and 120 min were 19.7±3.0%, 16.6±2.6%, 17.5±1.20%, and 15.4±0.35%, respectively, and those of the CoQ_10_-pretreated cells were 4.3±0.5%, 3.0±0.1%, 14.7±0.5%, and 12.9±0.4%, respectively. EtOH exposure caused a significant change in the mitochondrial membrane potential (30 min, *P* = 0.03; 60 min, *P* = 0.02; 90 min, *P* = 0.03; 120 min, *P* = 0.03). Pretreatment with CoQ_10_ significantly reduced MPT in the corneal fibroblasts at 30 (*P*<0.001), 60 (*P*<0.001), 90 (*P* = 0.04), and 120 min (*P* = 0.02).

### Effects of coenzyme Q_10_ on ethanol-treated cellular caspase activity

**Figure 5:**
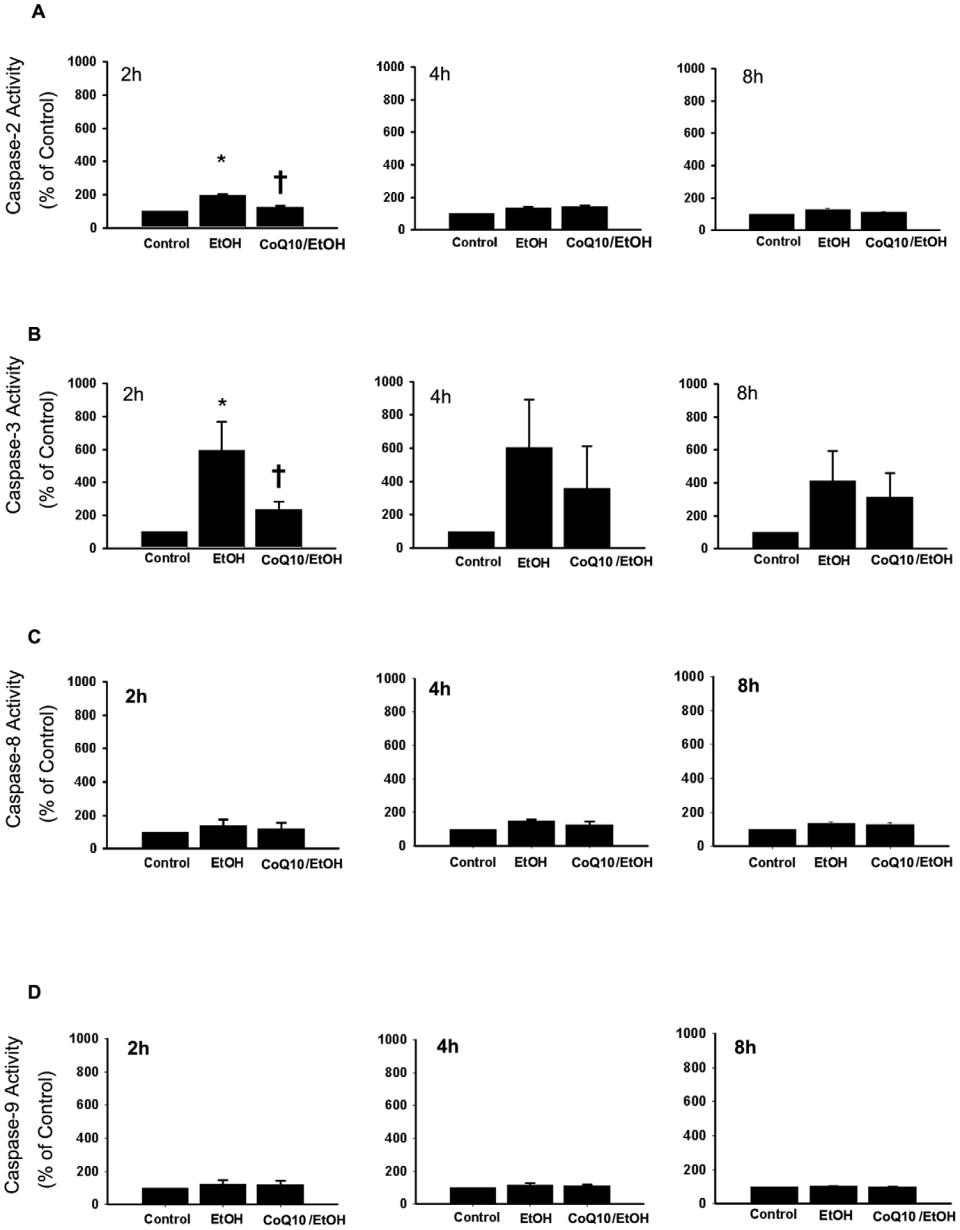
Pretreatment of corneal fibroblasts for 2 h with 10 µM CoQ_10_ prevented the activation of caspase 2 and caspase 3 induced by EtOH exposure. The activity of (A) caspase 2, (B) caspase 3, (C) caspase 8, and (D) caspase 9 in cells pretreated with or without CoQ_10_, followed by EtOH treatment (20%, 20 s), was examined by colorimetric assay after intervals of 2, 4, and 8 h. Data represent the results of three independent experiments performed in triplicate (means ± SD; *P<0.05 compared with the control group; †*P*<0.05 compared with the EtOH group).

To ascertain the effects of CoQ_10_ on the EtOH-induced activation of caspases (caspase 2, caspase 3, caspase 8, and caspase 9), cytosolic lysates of cells pretreated with or without CoQ_10_ and then exposed to EtOH (20%, 20 s) were used to measure the catalytic activation of the caspases at 2, 4, and 8 h (Figure 5). In the EtOH-exposed cells, the enzymatic activation of caspase 2 and caspase 3 began 2 h after treatment. There was an increase of about sixfold and twofold in caspase 3 and caspase 2 activity, respectively, 2 h after EtOH exposure. CoQ_10_ pretreatment significantly reduced the activation of caspase 2 and caspase 3 at 2 h after EtOH exposure (caspase 2, *P* = 0.01; caspase 3, *P* = 0.03). In contrast, the activities of caspase 8 and caspase 9 were not significantly different with or without CoQ_10_ pretreatment at 2, 4, and 8 h after EtOH exposure.

### Effects of coenzyme Q_10_ on ethanol-treated cellular caspase expression

**Figure 6:**
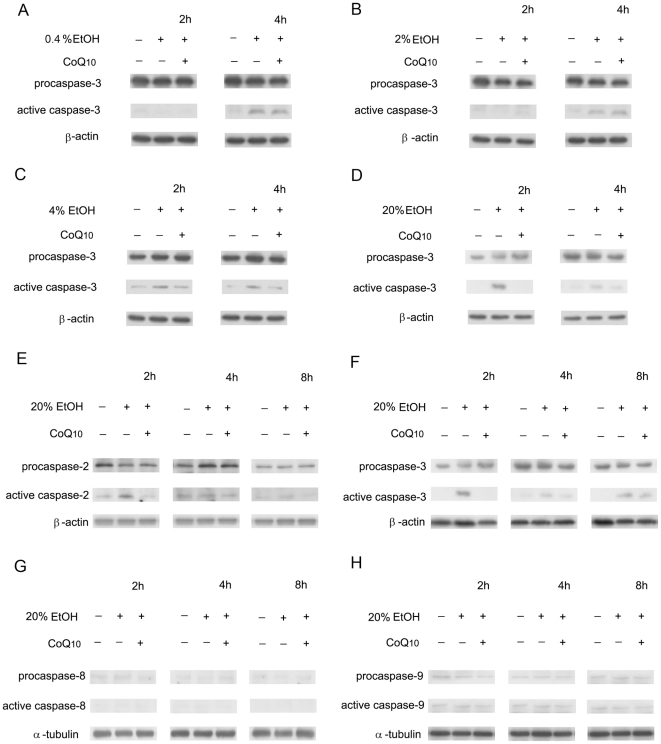
The effect of pretreatment of corneal fibroblasts with CoQ_10_ on the expression of active caspases. The cells were pretreated with 10 µM CoQ_10_ for h and thereafter not exposed or exposed to different concentrations of EtOH (A: 0.4%, B: 2%, C: 4%, D–H: 20%) for 20 s. Western blot analysis was performed after intervals of 2, 4, or 8 h. The panels showed representative blots of pro- and active forms of (A–D, F) caspase 3, (E) caspase 2, (G) caspase 8, and (H) caspase 9. β-Actin and α-tubulin were used as the loading controls.

To confirm the inhibition of caspase activation by CoQ_10_ pretreatment, a western blot analysis was performed (Figure 6). In the EtOH-exposed cells, the expression of active caspase 3 was evident at 4 h in 0.4% and 2% EtOH group, at 2, 4 h in 4% and 20% EtOH groups (Figure 6A–6D). Pretreatment with CoQ_10_ decreased the expression of active caspase 3 (Figure 6A–6D). In 20% EtOH-exposed cells, the expression of active caspase 2 and active caspase 3 was increased 2 h after treatment (Figure 6E, 6F). CoQ_10_ pretreatment significantly reduced the expression of active caspase 2 and active caspase 3 at 2 h after EtOH (20%) exposure (caspase 2, *P* = 0.01; caspase 3, *P* = 0.02; Figure 6E–6F). The expression of caspase 8 and caspase 9 did not differ at the indicated time points with or without CoQ_10_ pretreatment (Figure 6G–6H).

## Discussion

The apoptosis of cultured corneal fibroblasts has been detected dose- and time-dependently after treatment with dilute ethanol [11]. Consistent with previous results, we also report the apoptogenic effects of ethanol on cultured corneal fibroblasts. In our study, apoptotic features were apparent in ethanol-treated cells and could be demonstrated as cell membrane disruption, detected with Annexin V staining. We found the phenomenon of EtOH-induced apoptosis occurred in different concentrations of EtOH treatment (ranging from 0.4% to 20%). While, in the presence of CoQ_10_, the proportion of apoptotic cells decreased significantly. CoQ_10_ exhibited the most remarkable protection in the cells exposed to 20% EtOH. We also determined the potential mechanisms of CoQ_10_ in modulating the apoptogenic pathways of EtOH on corneal fibroblasts in vitro. These results show that CoQ_10_ reduced mitochondrial depolarization, ROS production, and the activation of caspase 2 and caspase 3 in corneal fibroblasts after EtOH treatment. Our results suggest that CoQ_10_ plays an antiapoptotic role under circumstances wherein corneal fibroblasts are challenged with an external stressor, such as ethanol. This protective effect was clearly demonstrated as increased cellular viability and reduced cellular apoptosis.

In clinical practice of corneal delamination with ethanol to remove corneal epithelium before PRK and LASEK, EtOH (20%) is exposed to the cornea. Joo et al. have reported the morphological and functional changes in the rat cornea after 20% EtOH exposure for 30 to 40 seconds. They found that the number of central stromal cells decreased gradually after EtOH exposure, meaning the corneal stromal cells did undergo cell death or apoptosis [33]. Similarly, Lee et al. have observed TUNEL-positive cells in the chick cornea stroma 4 h after 20% EtOH exposure for 30 seconds. Transmission electron microscopy showed similar evidence of apoptosis in the anterior stroma [34]. In addition, percutaneous injection of different concentrations of ethanol (0.4–100%) has been proven to be effective for the treatment of hepatocellular carcinoma by killing the hepatic cells [35]. Clinical and experimental assessment has shown that the low concentrations of ethanol (0.2–0.4%) effectively destroy hepatic cells by inducing apoptosis [35], [36]. According to these studies, we explored the phenomenon of EtOH-induced cell apoptosis at different concentrations of dilute EtOH (ranging from 0.4% to 20%) in cornea. Similarly, we revealed that EtOH-induced cell apoptosis could be found at these concentrations. Although the concentrations of the penetrating alcohol were uncertain, it is possible that alcohol leaks through the basement membrane and causes the stromal cells to undergo apoptosis.

The first stage of stromal wound healing in the cornea after PRK and LASEK is stromal cell apoptosis, followed by stromal cell proliferation and subsequent myofibroblast transformation [6], [7]. Therefore, to achieve uneventful wound healing and optimal visual recovery, it is important to prevent the initiation of keratocyte apoptosis during refractive surgery [8]. In our study, we demonstrated that application of EtOH did contribute to the apoptosis of corneal fibroblasts in corneal stroma, which might further affect the wound healing process *in vivo*.

In the apoptotic signaling cascades, two different initiation machineries play major roles in a variety of cell types: extrinsic death-receptor-mediated signaling and intrinsic caspase family cysteine-protease-mediated signaling [37], [38]. The Fas/Fas L and caspase 3 apoptotic pathway is thought to play a primary role in acute ethanol-induced liver apoptosis in mice [39], [40]. Pastorino *et al* have demonstrated that mitochondrial depolarization and the subsequent activation of the caspase-independent pathway occur in ethanol-exposed hepatocytes [41]. In cerebellar granule neurons, ethanol exposure induces strong increases in caspase 2, 3, 6, 8, and 9 activities [42]. In the present study, we found that caspase 3 plays a critical role in the ethanol-induced apoptosis of corneal fibroblasts [10], [11]. We also demonstrated the inhibitory effects of CoQ_10_ on caspase 3 and caspase 2, but not on caspase 8 or caspase 9. Here, the notion that ethanol induces apoptosis by different pathways, depending on the cell type, can also be extended to the apoptosis of corneal fibroblasts. The specific roles of mitochondrial depolarization, oxidative stress, and caspase activation after ethanol exposure warrant further investigation and comparison in different cell types.

Mitochondria play major roles in cellular physiology beyond their roles in electron transport and energy conservation. The outer membrane and the intermembrane space, considered mere accessories in classical mitochondrial physiology, are now recognized as intimately involved in the control of apoptosis, the process of controlled (programmed) cell death [43]. There is now considerable evidence that ethanol treatment facilitates the activation of MPT [43], [44], [45]. Ishii and coworkers reported that acute ethanol treatment promotes apoptosis in primary hepatocyte cultures *in vitro*, accompanied by ROS formation and mitochondrial depolarization, characteristic of MPT activation [45]. Mitochondrial dysfunction in apoptotic signaling after exposure to ethanol has not been specifically addressed in the context of the apoptosis of corneal fibroblasts, although altered mitochondrial function has been reported in the corneal epithelium [10]. Our study demonstrates for the first time that mitochondrial dysfunction in corneal fibroblasts occurs soon after ethanol exposure. CoQ_10_ exerts its antiapoptotic effects by abrogating mitochondrial disruption in ethanol-treated cells. We propose that the potential mechanism by which CoQ_10_ inhibits mitochondrial dysfunction is associated with the opening of the mitochondrial permeability transition pore (PTP). Previous studies have demonstrated that the Ca^2+^-dependent opening of the PTP in isolated mitochondria can be prevented by two synthetic quinine analogues [46], [47], and the structural features required for the regulation of the mitochondrial PTP by ubiquinone analogues have been identified [48]. CoQ_10_ is also reported to prevent the mitochondrial depolarization and cytochrome *c* release induced by apoptotic stimuli [49]. All these sequential events are related to the opening of the PTP, and lead to mitochondria-dependent apoptosis.

Regarding ethanol-induced ROS formation, increased cellular oxidative stress occurs during the acute and chronic ethanol exposure of hepatocytes [50], the developing brain [51], and cultured neurons [52]. Ethanol elicits a rapid increase in ROS, which is ultimately followed by apoptotic cell death in cultured neurons [53]. The proposed mechanisms of ethanol-induced ROS formation include oxidative protein modification and enzyme inactivation [54], antioxidant depletion [55], and mitochondrial dysfunction [51], all of which may contribute to apoptotic cell death [53]. It has been proposed that the inhibition of ROS production by CoQ_10_ and other ubiquinones is mediated by the regulation of mitochondrial depolarization [48]. Consistent with previous studies, we found that the CoQ_10_ prevents both mitochondrial depolarization and ROS production in EtOH-exposed cells at similar time points. Based on these considerations, CoQ_10_ plays an important role in modulating mitochondrial depolarization and ROS production after the acute exposure of cells to ethanol.

The management and response systems for cellular oxidative stress involve a cascade of carefully maintained homeostatic processes that depend on mitochondrial function after ethanol treatment. The mitochondria are one of the important sources of ROS in ethanol-exposed cells [50]. The resulting oxidative stress in the mitochondria exerts its effects in part by damaging the mitochondrial functions. The mitochondrial response is further controlled by the activation of the MPT, and changes in the mitochondrial potential induce caspase activation. Finally, the consequences of mitochondrial depolarization lead to programmed cell death. In this study, we found that the response systems after ethanol treatment are affected by CoQ_10_ at different levels. CoQ_10_ can suppress the oxidative stress imposed on the mitochondria and thereby suppresses the MPT. CoQ_10_ also prevents the activation of caspases and subsequent apoptosis. Our finding that CoQ_10_ protects cells from mitochondria-dependent apoptosis is consistent with those of one previous study [49]. The association and integration of CoQ_10_ in the mitochondrial respiratory chain complexes suggest that CoQ_10_ plays a role as a structural element and modulator of the mitochondria [32], [46], [47], [48], [49].

In the present study, we have demonstrated that CoQ_10_ exerts a potent protective effect against ethanol-induced programmed cell death by inhibiting mitochondrial depolarization and reducing intracellular oxidative stress, caspase activity, and subsequent apoptosis. Our results not only offer a more mechanistic explanation of the antiapoptotic properties of CoQ_10_, but also support further investigation of the clinical uses of CoQ_10_ in treating and preventing the cell damage in the cornea induced by ethanol exposure.

## Materials and Methods

### Culture of corneal fibroblasts

Primary corneal fibroblasts were obtained from fresh bovine corneas by collagenase digestion, with a method modified from that described by Funderburgh *et al* [56]. Briefly, the central portions of fresh bovine corneas were incubated at 37°C in 2.4 U of dispase II (Roche, Penzberg, Germany)/Dulbecco's modified Eagle's medium (DMEM; Invitrogen-Gibco, USA) containing antibiotics (penicillin, 50 µg/mL; streptomycin, 50 µg/mL; amphotericin B, 2.5 µg/mL) for 3 h to remove the corneal epithelium and endothelium. After dispase II digestion, the samples were serially scraped with a plastic spatula (Cell Scraper, TPP, Switzerland) in phosphate-buffered saline (PBS) to remove the epithelial cells. The corneal endothelial cells and Descemet's membrane were peeled away in a sheet from the periphery to the center of the inner surface of the cornea with fine forceps.

The tissue was rinsed twice with DMEM containing antibiotics. It was then minced into several small parts (2–3 mm) and incubated in a volume of 1 mL per corneal stoma of 2 mg/mL (w/v) collagenase A (103-586, Roche, Penzberg, Germany) in DMEM with antibiotics at 37°C for 12 h until the complete disruption of the tissue. Nylon mesh (40 mm; Cell Strainer, Falcon, USA) was used to filter the cell suspension. The filtered cell suspension was incubated in 75 mL flasks at 37°C with 10% fetal bovine serum (FBS; Invitrogen-Gibco) in 95% air/5% CO_2_. The samples were serially trypsinized and passaged three times for the experiments.

### Treatment

Treatment with 10 µM CoQ_10_ dissolved in 0.04% Lutrol F217, which was used as the vehicle to ensure the cellular uptake of this hydrophobic molecule [49], was commenced 2 h before the application of EtOH (Figure S1). Corneal fibroblasts cultured to approximately 90% confluence were pretreated with or without CoQ_10_ and then exposed to EtOH (0.4, 2, 4, and 20%) for 20 s. EtOH was diluted in distilled water to yield indicated concentrations of EtOH solution. Cells in the control group were treated with medium only.

### MTT assay

Cell viability was analyzed using the 3-(4,5-dimethylthiazol-2-yl)-2, 5-diphenyltetrazolium bromide (MTT) assay. Cells (4 × 10^4^/well) were grown overnight in 96-well plates, pretreated with or without CoQ_10_, and then exposed to EtOH (0.4, 2, 4, and 20%) for 20 s. After different incubation periods (2, 4, 8 h, or 12 h), MTT (0.5 mg/mL) was added to each well. The samples were incubated in the absence of light for 4 h, after which the medium was removed. The precipitates were resuspended in 50 µL of DMSO. The absorbance was measured on a plate reader at 570 nm (or 540 nm). Each experiment was performed in triplicate. The reduction in optical density caused by the EtOH treatment was used as a measure of cell viability after it was normalized to that of cells incubated in control medium, which were considered 100% viable [57].

### Identification of apoptosis induced by ethanol

To examine the apoptosis in EtOH-exposed (0.4–20%, 20 s) cells with or without 2 h of CoQ_10_ pretreatment, the cells were washed twice with PBS and then incubated for 4, 8, or 12 h. The cells were simultaneously subjected to annexin V and propidium iodide (PI) assays. An annexin V–fluorescein isothiocyanate (FITC) apoptosis detection kit (Serotec, Oxford, UK) was used to bind phosphatidylserine, which is translocated to the outer leaflet of the plasma membrane during the early stages of cell apoptosis [58]. Therefore, the apoptotic cells were only stained with annexin V–FITC, whereas the necrotic cells were doubly stained for both annexin V–FITC and PI. The cells were suspended in binding buffer at a final cell concentration of 1×10^5^ cells/mL and incubated with both annexin V–FITC and PI for 15 min in the dark. The exposed phosphatidylserine was measured by fluorescence-activated cell sorter analysis.

### Determination of reactive oxygen species (ROS)

Intracellular ROS were measured based on the intracellular peroxide-dependent oxidation of 2′,7′-dichlorodihydrofluorescein diacetate (DCFH-DA; Molecular Probes, USA) to form the fluorescent compound 2′,7′-dichlorofluorescein (DCF), as previously described [59]. The ROS levels were assessed early after ethanol exposure, before the occurrence of apoptosis, as examined by flow cytometry. The cells were seeded onto 48-well plates at a density of 2×10^4^ cells/well and cultured for 48 h. After the cells were washed twice with PBS, fresh medium with or without 10 µM CoQ_10_ was added. The cells were incubated for 2 h and then exposed to EtOH (20%, 20 s). DCFA-DA (20 µM) was added to the cells, which were incubated for 30 min at 37°C. After 30, 60, 90, and 120 min, the cells were harvested and resuspended in 50 mM HEPES buffer (5 mM HEPES, [pH 7.4], 5 mM KCl, 140 mM NaCl, 2 mM CaCl_2_, 1 mM MgCl_2_, and 10 mM glucose). The fluorescence intensity was determined spectrofluorometrically (Geminin EM), with excitation at 485 and emission at 530 nm.

### Measurement of changes in mitochondrial membrane potential (ΔΨm)

To determine ΔΨm, the cells were pretreated with or without CoQ_10_ and then exposed to EtOH (20%, 20 s). After 30, 60, 90, or 120 min, 2 µM 5,5′,6,6′-tetrachloro-1,1′,3,3′-tetraethyl-benzimidazolyl-carbocyanine iodide (JC-1; Molecular Probes, USA) was added to the cells for 30 min. After the JC-1 was removed, the cells were washed with PBS, harvested by trypsinization, and resuspended in PBS. The amount of JC-1 retained by 10,000 cells per sample was measured at 530 nm (green fluorescence) and 590 nm (red fluorescence) with a flow cytometer and analyzed with Cell Quest Alias software. The changes in green fluorescence were evaluated to determine ΔΨm [60].

### Caspase activity assay

To measure the levels of caspase activity, cells pretreated with or without CoQ_10_ were exposed to EtOH (20%, 20 s). The caspase levels were measured with Caspase-3/CPP32, Caspase-2, Caspase-8, and Caspase-9 Colorimetric Assay Kits (BioVision, Palo Alto, CA) at 2, 4, and 8 h after EtOH exposure. The cells were resuspended in 100 µL of chilled lysis buffer and incubated on ice for 10 min. After incubation, the cells were centrifuged for 1 min in a microcentrifuge (10,000 × g). The supernatant (cytosol extract) was transferred to a fresh tube and left on ice. After assessment of the protein concentration (bicinchoninic acid [BCA] method), each cytosolic extract was diluted to a concentration of 50–200 µg protein/50 µL cell lysis buffer (1–4 mg/mL). The samples were measured and aliquoted to provide a 2 × reaction buffer into a glass tube (assuming 50 µL of 2 × reaction buffer per sample). A volume of 2 × reaction buffer containing 10 mM DTT (50 µL) and 5 µL of the appropriate caspase substrate (4 mM) was added to each sample (final concentration 200 µM). The substrates used for the different caspases were DEVD-pNA for caspase 3, VDVAD-pNA for caspase 2, IETD-pNA for caspase 8, and LEHD-pNA for caspase 9. The samples were incubated for 2 h in the dark. The absorbance was measured on a plate reader at 400 nm (or 405 nm).

### Western blot analysis

For the western blot analysis, the corneal fibroblasts were treated with or without CoQ_10_ and then exposed to EtOH (0.4, 2, 4, and 20%) for 20 s. After 2, 4, or 8 h, the cytosolic fractions were prepared as reported previously [61]. The total protein concentration of each sample was measured using the BCA method. The total cell protein (40 µg) was resolved by SDS–PAGE on 12% acrylamide gels and blotted onto polyvinylidene difluoride membrane. The blotted membrane was incubated with PBS/Tween 20 (0.05%) solution containing 2% skim milk to block nonspecific antigens, and then incubated with rabbit anti-human caspase 3, caspase 8, or caspase 9 antibody (Cell Signaling, Denvers, MA) or rabbit anti-human/mouse caspase 2 antibody (R&D Systems, Minneapolis, MN) at room temperature for 1 h. After the primary antibody reaction, the membranes were incubated with horseradish-peroxidase-conjugated anti-rabbit IgG antibody (Cell Signaling), detected by Chemiluminescence Reagent Plus (NEN, Boston, MA), and exposed to film (BioMax MR, Kodak).

### Statistical analysis

Values are expressed as means ± SD. The statistical analysis was performed with Student's *t* test and one-way analysis of variance (ANOVA) where appropriate. Statistical significance was determined at the 0.05 level.

## Supporting Information

Figure S1 **10 μM of CoQ_10_ was the optimal concentration to prevent cell death after EtOH exposure.** The cells was pretreated with different concentration of CoQ_10_ (0, 1, 2, 5, 10, 20, and 40 μM) followed by EtOH exposure (20%, 20 s). The cell viability was evaluated by MTT assay at 2, 4, 8, and 12 h. The maximal cell viability was found when the cells were pretreated with 10 μM of CoQ_10_ at all time points. Data represent the results of three independent experiments performed in triplicate (means ± SD; *P < 0.05).
(TIF)
