# Supplementary figures and images for: Coenzyme Q10 Reduces Ethanol-Induced Apoptosis in Corneal Fibroblasts
Source: PLoS One. 2011 Apr 27;6(4):e19111. doi: 10.1371/journal.pone.0019111 (PMC3083429; doi:10.1371/journal.pone.0019111)

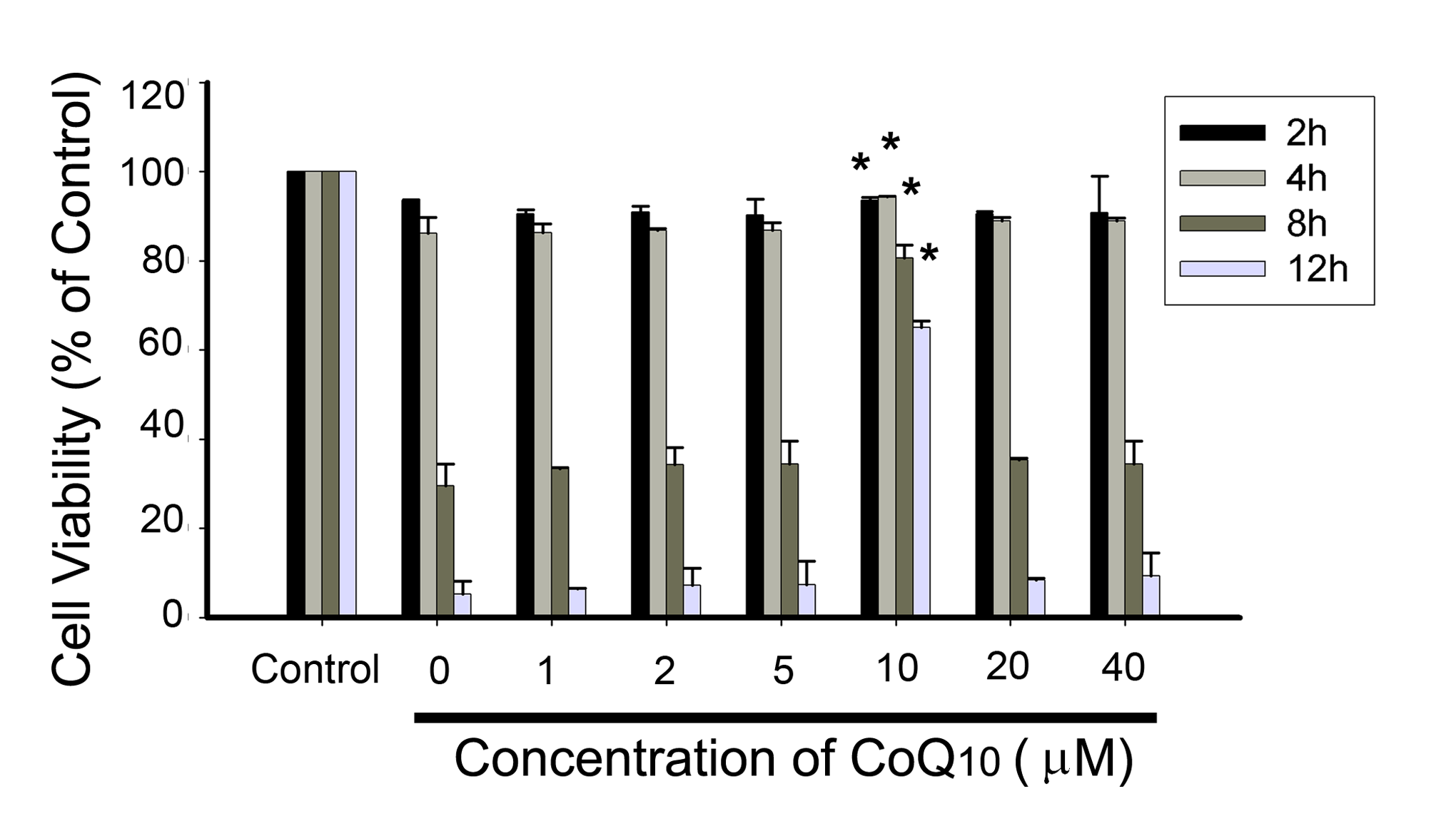

Supplement: Figure S1 — 10 μM of CoQ10 was the optimal concentration to prevent cell death after EtOH exposure. The cells was pretreated with different concentration of CoQ10 (0, 1, 2, 5, 10, 20, and 40 μM) followed by EtOH exposure (20%, 20 s). The cell viability was evaluated by MTT assay at 2, 4, 8, and 12 h. The maximal cell viability was found when the cells were pretreated with 10 μM of CoQ10 at all time points. Data represent the results of three independent experiments performed in triplicate (means ± SD; *P < 0.05). (TIF) [file pone.0019111.s001.tif]
